# Effect of fenugreek (*Trigonella foenum-graecum* L.) intake on glycemia: a meta-analysis of clinical trials

**DOI:** 10.1186/1475-2891-13-7

**Published:** 2014-01-18

**Authors:** Nithya Neelakantan, Madanagopal Narayanan, Russell J de Souza, Rob M van Dam

**Affiliations:** 1 Saw Swee Hock School of Public Health, National University of Singapore and National University Health System, Singapore, Singapore; 2 Department of Medicine, Yong Loo Lin School of Medicine, National University of Singapore and National University Health System, Singapore, Singapore; 3 Department of Clinical Epidemiology & Biostatistics, McMaster University, Hamilton, ON, Canada; 4 Clinical Nutrition and Risk Factor Modification Center, St. Michael’s Hospital, Toronto, ON, Canada; 5 Department of Nutrition, Harvard School of Public Health, Harvard University, Boston, MA, USA

**Keywords:** Fenugreek, Trigonella, Nutrition, Glycemia, Diabetes management, Clinical trials, Systematic review, Meta-analysis

## Abstract

**Background and aim:**

Fenugreek is a herb that is widely used in cooking and as a traditional medicine for diabetes in Asia. It has been shown to acutely lower postprandial glucose levels, but the long-term effect on glycemia remains uncertain. We systematically reviewed clinical trials of the effect of fenugreek intake on markers of glucose homeostasis.

**Methods:**

PubMed, SCOPUS, the Cochrane Trials Registry, Web of Science, and BIOSIS were searched up to 29 Nov 2013 for trials of at least 1 week duration comparing intake of fenugreek seeds with a control intervention. Data on change in fasting blood glucose, 2 hour postload glucose, and HbA1c were pooled using random-effects models.

**Results:**

A total of 10 trials were identified. Fenugreek significantly changed fasting blood glucose by -0.96 mmol/l (95% CI: -1.52, -0.40; I^2^ = 80%; 10 trials), 2 hour postload glucose by -2.19 mmol/l (95% CI: -3.19, -1.19; I^2^ = 71%; 7 trials) and HbA1c by -0.85% (95% CI: -1.49%, -0.22%; I^2^ = 0%; 3 trials) as compared with control interventions. The considerable heterogeneity in study results was partly explained by diabetes status and dose: significant effects on fasting and 2 hr glucose were only found for studies that administered medium or high doses of fenugreek in persons with diabetes. Most of the trials were of low methodological quality.

**Conclusions:**

Results from clinical trials support beneficial effects of fenugreek seeds on glycemic control in persons with diabetes. However, trials with higher methodology quality using a well characterized fenugreek preparation of sufficient dose are needed to provide more conclusive evidence.

## Introduction

The prevalence of diabetes mellitus is increasing worldwide with approximately half of all persons with diabetes living in Asia [1]. The herb fenugreek (*Trigonella foenum-graecum* L., Fabaceae family) is used both in cooking and for the treatment of diabetes in many parts of the world, especially in China, Egypt, India and Middle Eastern countries [2-4]. In low-income countries, individuals with diabetes often do not have access to appropriate medications due to a lack of financial resources [5]. Active compounds of fenugreek included soluble fiber [6-8], saponins [9,10], trigonelle [11], diosgenin [12], and 4-hydroxyisoleucine [13,14]. Hypoglycemic activities have mainly been attributed to dietary fiber [6,7] and saponin [9]. Fenugreek is a widely used herbal medicine for diabetes, but its efficacy for glycemic control remains unclear.

Animal studies have shown that fenugreek seed extracts have the potential to slow enzymatic digestion of carbohydrates, reduce gastrointestinal absorption of glucose, and thus reduce post-prandial glucose levels [8]. In addition, fenugreek stimulated glucose uptake in peripheral tissues [15] and had insulinotropic properties in isolated rat pancreatic cells [16]. In humans, fenugreek seeds acutely reduced postprandial glucose and insulin levels [17-20]. In addition, several longer-term clinical trials showed reductions in fasting and post-prandial glucose levels and glycated haemoglobin (HbA1c) [9,21-23], but some trials did not show benefit [24,25]. Systematic reviews that have evaluated the effect of various alternative therapies for diabetes included only a few clinical trials of fenugreek [26-29]. We therefore conducted a systematic review and meta-analysis of the effects of fenugreek on glucose homeostasis based on a comprehensive literature search leading to the identification of a reasonably large number of trials with an evaluation of potential explanations for differences in study results.

## Methods

### Data sources and searches

To identify articles on the effect of fenugreek on glucose homeostasis we searched MEDLINE (PubMed), SCOPUS, Web of Science, BIOSIS, and Cochrane Trials Registry from inception through Nov 29, 2013 using key search terms related to fenugreek (“fenugreek”, “trigonella”), an experimental study design (“trial”, “clinical trial”, “intervention”, “therapy”), to identify potentially relevant articles. The search strategy utilized both index terms and free text to search for synonyms of trigonella, fenugreek and diabetes/healthy subjects, and was limited to human studies. Grey literature such as conference proceedings, abstracts, dissertations and technical reports was identified using the same key terms through the electronic search engines Google Scholar, SCIRUS, CINAHL, and ProQuest. No language restriction was applied.

The results (titles, abstracts and citations) of electronic searches were downloaded into EndNote software (EndNote X5, 2011, Thomson Reuters, Philadelphia) and initial screening for eligibility was performed by two independent reviewers (Nithya Neelakantan, Madanagopal Narayanan). When assessment of eligibility based on the title and abstract was insufficient, the full text of the articles was obtained. The second screening of those full text articles was then independently performed by at least two reviewers (Nithya Neelakantan, Madanagopal Narayanan, Rob M van Dam). Disagreements were resolved by consensus. The kappa for the inter-reviewer reliability was 0.78. Study authors were contacted to verify results and methodological quality of retrieved articles where necessary. We used the Preferred Reporting Items for Systematic Reviews and Meta-Analysis (PRISMA) statement to report our findings [30].

### Study selection

**Figure 1:**
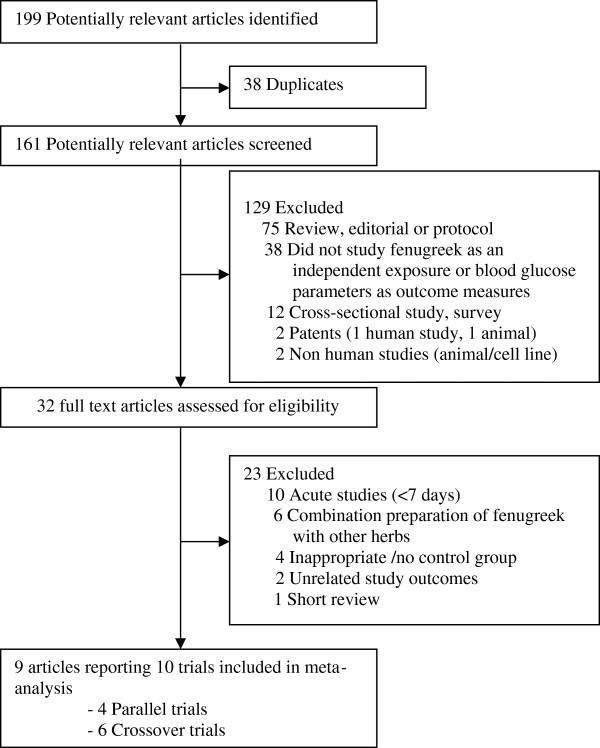
**Selection of trials for meta-analysis.** (Search was conducted to identify articles up to 29 Nov 2013).

We included clinical trials that compared single herb preparations of fenugreek in any dose or form with a control intervention that was either placebo or no treatment and evaluated effects on markers of glycemia [fasting blood glucose, 2 hr postload glucose, glycosylated hemoglobin (% HbA1c) and/or fasting serum insulin levels]. We excluded trials that used combination preparations of fenugreek with other herbs, non-human studies, observational studies, literature reviews/editorials/letters/case reports, and articles not reporting the outcomes of interest. We also excluded trials with interventions that lasted less than 7 days. The number of articles that did not meet the eligibility criteria and the reasons for their exclusion are shown in Figure 1.

### Data extraction and quality assessment

Details of trial design, study setting, population, randomization, blinding, sample size, duration of follow-up, participant characteristics, interventions, total daily dose and outcome characteristics were independently extracted by two reviewers (Nithya Neelakantan, Madanagopal Narayanan), using a standardized data extraction form. Differences in data extraction were resolved by a third reviewer (Rob M van Dam). The quality assessment was conducted using the CONSORT statement for herbal trials [31] by two reviewers (Nithya Neelakantan, Madanagopal Narayanan), with disagreements resolved by consensus.

From each trial, data on mean and SD for all outcomes of interest were extracted. If trials reported fasting blood glucose and 2 hr postload glucose (glucose concentrations 2 hours after the start of the oral glucose tolerance test) in units of mg/dL, this was converted to the standardized international unit [32] of mmol/L by multiplying the glucose values in mg/dL by 0.0555; for fasting serum insulin, we divided the serum insulin values reported in pmol/L by 6.945 and reported the results in mU/L.

Parallel trials generally reported the baseline mean and standard deviation and follow-up mean and standard deviation, but not the standard deviation (SD) of change for the intervention and control groups. For parallel trials, the net changes in each outcome measure were calculated as the change in the intervention group minus the change in the control group. For crossover trials, net changes in the outcome measures were calculated as the value of the outcome measure at the end of the intervention period minus the value of the outcome measure at the end of control period. We estimated the SD of the change on the basis of reported p values for differences in means, if available [33]. We used the p-values cutoff if it was only reported that a p-value was below a threshold (e.g., 0.05 if p < 0.05 was reported) leading to conservative estimates [34,35]. If p-values were not reported, we imputed SD of the change by using a pooled correlation coefficient between baseline and final measurements from a meta-analysis of correlation coefficients from those trials reporting sufficient data. We derived correlation coefficients for individual trials according a standard formula [33] and we then imputed these correlations into the meta-analysis as transformed z scores (±SEs) to estimate the pooled correlation coefficient [36]. For HbA1c and fasting serum insulin measures, due to small number of trials, we estimated the SDs of the change assuming a conservative 0.5 correlation and performed a sensitivity analysis assuming alternative values of 0.25 or 0.75. To investigate the effect of imputed within-person correlation coefficients, we performed sensitivity analyses with a range of correlation coefficients (0.25, 0.50 and 0.75) [37], the pooled estimates did not change substantially.

### Meta-analysis

The meta-analysis was performed according to the methods described by Curtin et al. [38]. In the combined design meta-analysis, the pooled estimate of treatment effect combining parallel and crossover trial results was the weighted sum of the separate treatment effects estimated, respectively, from parallel and crossover trials divided by the sum of the associated weights. We anticipated large differences in the fenugreek drug preparation format, active components/chemical composition, administration of supplements, and dosages as well as variation in the study population and study design. Therefore, we *a priori* decided to use a random effects model for this meta-analysis. Hence, for each outcome measure, weighted mean differences and corresponding 95% confidence intervals (CI) were calculated by using DerSimonian and Laird random-effects models. We also conducted separate meta-analyses for parallel and crossover trials for the primary outcome measures, fasting blood glucose, and 2 hr post prandial glucose.

Heterogeneity in study results was tested by using the Cochran *Q* statistic (and associated p value), and was quantified by the *I*^2^ statistic. The *I*^*2*^ provides an estimate of the percentage of variation in study results that is explained by between-study heterogeneity rather than sampling error [39]. Potential sources of heterogeneity were investigated using *a priori* defined stratified analyses by study design (parallel or crossover), daily dose of fenugreek extract (<5 g, 5–10 g or >10 g), study duration (<30 days or > =30 days), randomization (yes or no), blinding (yes or no), baseline BMI (<25 or > =25 kg/m^2^), study precision (SE of the effect estimate above or below the median), geographical region (India vs. other countries) and age (above or below the median mean age of all studies).

Meta-regression analyses were used to assess the significance of differences in the effects of fenugreek between strata. p-values for the overall F-test for a common mean amongst three or more groups were obtained using one way ANOVA. Publication bias was investigated by visual inspection of funnel plots and by the Egger regression test and the Begg adjusted correlation test [40]. The robustness of the findings of the meta-analysis to different assumptions were examined in a sensitivity analysis using both fixed and random effects models, in which the meta-analysis estimates were computed omitting one trial at a time to assess the influence of each individual trial [41]. All tests were two-sided and p <0.05 was considered statistically significant. The data were analyzed by using Stata version 11 (StataCorp, College Station, Texas).

## Results

### Search results

We identified 161 potentially relevant articles and screened the abstracts for eligibility. The flow of trial selection is reported in Figure 1. We evaluated 32 full text articles in detail. Nine articles reporting 10 trials met the inclusion criteria for the meta-analysis. Of these 10 trials, one trial [42] reported results separately for participants with mild and severe type 2 diabetes, and thus 11 data points are presented in Table 1. Five of these had a parallel design and six had a crossover design.

**Table 1:** Characteristics of the 10 trials (11 data points) included in the meta-analysis of the effects of fenugreek seeds on glycemia

**Lead author, year and country**	**Population, medication**	**Design**	**Age, y**	**Male (%)**	**Sample size, N**	**Duration, days**	**Fenugreek, preparation**	**Daily dose, g**	**Control**	**Outcomes**	**Study quality**		
											**RCT**	**Blinding**	**Dropout**
Bordia et al., 1997 [42], India	Mild T2DM, NR	PL	NR	NR	40	30	Capsule, PS	5	Placebo	FBG, 2 hr glucose	NR	NR	NR
Bordia et al., 1997 [42], India	Severe T2DM, NR	PL	NR	NR	40	30	Capsule, PS	5	Placebo	FBG, 2 hr glucose	NR	NR	NR
Chevassus et al., 2010 [43], France	Overweight, NR	PL	38.0	100	40	42	Capsule, H	1.176	Placebo	FBG, FSI	Yes	DB	5%
Gupta et al., 2001 [23], India	T2DM, SU, BI	PL	51.0	76	25	56	Capsule, H	1	Placebo	FBG, 2 hr glucose, HbA1c, FSI	Yes	DB	4%
Lu et al., 2008 [9], China	T2DM, SU	PL	54.4	55	69	84	Capsule, PS	6.3	Placebo	FBG, 2 hr glucose, HbA1c	Yes	DB	NR
Alamdari et al., 2009 [17], Iran	T2DM, Diet, OAD	CO	43.1	100	12	56	PS	8	Unspecified	FBG, 2 hr glucose, HbA1c	Yes	NR	25%
Chevassus et al., 2009 [25], France	Healthy, NR	CO	22.0	100	12	14	Capsule, H	1.176	Placebo	FBG, FSI	Yes	DB	0%
Raghuram et al., 1994 [7], India	T2DM, BI	CO	46.6	NR	10	15	Chapati^a^, PS	25	Chapati	FBG, 1 hr glucose, HbA1c	Yes	NR	NR
Sharma et al., 1990 [44], India	T1DM, Insulin therapy	CO	22.7	70	10	10	Chapati^a^, DPS	100	Chapati	FBG, 2 hr glucose, FSI	Yes	NR	NR
Sharma et al., 1990 [22], India	T2DM, BI/metformin	CO	46.0	67	15	10	Chapati^a^, DPS	100	Chapati	FBG, 2 hr glucose, FSI	Yes	NR	NR
Sharma et al., 1990 [22], India	T2DM, NR	CO	42.0	NR	5	20	Chapati^a^, DPS	100	Chapati	FBG, 2 hr glucose	Yes	NR	NR

*Abbreviations*: *NR* not reported, *T1DM* type 1 diabetes mellitus, *T2DM* type 2 diabetes mellitus, *SU* sulfonylurea, *BI* biguanides, *OAD* oral antidiabetic drug, *PL* parallel, *CO* crossover trials, Daily dose, *g* fenugreek total daily dose in grams, *PS* powdered fenugreek seeds, *H* Hydro-alcoholic extract of fenugreek seeds, *DPS* Debitterized fenugreek seed powder, *FBG* fasting blood glucose, *HbA1c* % glycosylated hemoglobin, *FSI* fasting serum insulin, *RCT* randomized control trial, *DB* double blinded, ^a^Fenugreek incorporated into chapati (unleavened bread).

### Trial characteristics

The mean age of participants in the trials ranged from 22.0 to 54.4 years (median: 43.1 y), and the median percentage of males was 76%. Most trials included participants with type 2 diabetes treated with diet or oral anti-diabetic medication (Table 1). One trial was conducted in persons with type 1 diabetes and two trials included overweight or non-overweight participants without diabetes. The sample size ranged from 5 to 15 participants for crossover trials and from 25 to 69 participants for parallel trials. The sample size for all trials combined was 278. The daily dose of fenugreek seed ranged from 1 g to 100 g (median: 25 g), and the study duration from 10 to 84 days (median: 30 days). Fenugreek supplements were administered as powdered fenugreek seeds, debitterized powdered fenugreek seeds, or hydro-alcoholic seed extract either in form of capsules or as an ingredient of unleavened bread. These were provided in equal doses 2 to 3 times per day. All 10 trials [7,9,17,22,23,25,42-44] (11 data points) reported fasting blood glucose, 7 trials [7,9,17,22,23,42,44] (8 data points) reported 2 hr glucose, 5 trials [22,23,25,43,44] reported fasting serum insulin and 3 trials [9,17,23] reported HbA1c. With regard to study quality, one trial [42] did not report whether groups were randomized, none of the trials reported details on the method of randomization or allocation concealment, and most trials [7,17,22,42,44] did not report the blinding status. Only four trials reported the percentage of drop-outs [17,23,25,43]. Of which three studies [23,25,43] have reported low percentage drop-out ranging between 0 to 5% and one study [17] has reported 25% drop-out. These subjects were not included in the statistical analyses that were conducted for the primary studies. Most trials provided information on the concentration of components of the used fenugreek preparations. Reported components included diosgenine, saponins, trigonelline (1.4%) and 4-hydroxyisoleucine (1.5%) for the hydro-alcoholic extract [25,43]; alkaloids, carpaine, erythricine, trigonelline, meletin, and saponins for powdered fenugreek seeds [22,44]; and lipids (0.1%), protein (28.3%), starch (6.5%), total fiber (51.7% [gum 19.2%]) for debitterized fenugreek seeds [22,44].

### Effect on glucose homeostasis

#### Fasting blood glucose

**Figure 2:**
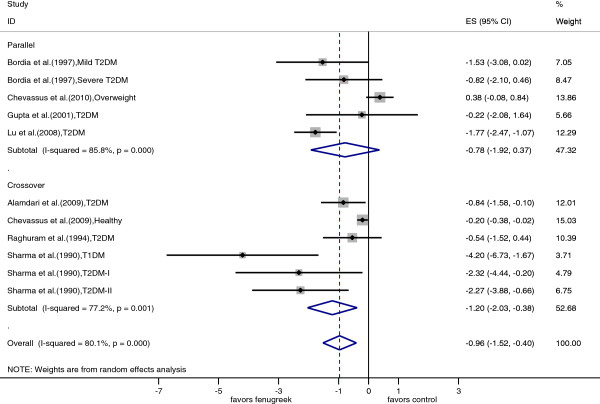
**Forest plot of the effect of fenugreek on fasting blood glucose.** The effects in individual trials are depicted as open squares with 95% confidence intervals (CIs). Pooled estimates with 95% CIs are depicted as open diamonds.

All 10 trials (11 data points) were included in the meta-analysis of fasting blood glucose. The individual trial results and the pooled estimates by trial designs are shown in Figure 2. Based on the overall pooled estimate, fenugreek significantly reduced blood glucose levels as compared with control treatments (pooled mean difference = -0.96 mmol/l; 95% CI: -1.52, -0.40; p = 0.001). There was large heterogeneity in study results (I^2^ = 80%; p < 0.001). There were no significant differences in the effects of fenugreek on fasting glucose by study design, study duration, geographical region, mean age and mean BMI of the study population (Table 2). However, the effect of fenugreek on fasting blood glucose differed significantly by diabetes status with substantial effects in persons with type 1 diabetes and type 2 diabetes, but not in persons without diabetes. There was a large variation in the dose of fenugreek used ranging from 1 g per day to 100 g per day of fenugreek seeds. The effect size differed significantly by the dose of fenugreek used suggesting no effects for the studies using low doses (<5 g/day) and greater effects with higher doses of fenugreek. Effects of fenugreek on fasting glucose also differed by preparation method of the fenugreek supplement. Studies using debitterized fenugreek powdered showed the greatest reduction in glucose levels, but these were mostly the same trials that administered the highest dose of fenugreek. Similarly, the trials using a hydro-alcoholic extract were the same trials that used the lowest dose. Heterogeneity in effects on fasting glucose was partly explained by fenugreek dose (I^2^_residual_ = 69%; adjusted R^2^ = 47%), fenugreek preparation method (I^2^_residual_ = 38%; adjusted R^2^ = 83%), and diabetes status of the study population (I^2^_residual_ = 55%; adjusted R^2^ = 61%).

**Table 2:** Stratified meta-analyses of the effects of fenugreek on fasting blood glucose and 2 hour postload glucose according to trial and participant characteristics

**Characteristics**	**Fasting blood glucose**					**2 hr-postload glucose**				
	**n**	**Pooled estimate**	**I**^**2 **^**(95% CI)**	**P**_**hetr**_	**P**_**EM**_	**n**	**Pooled estimate**	**I**^**2 **^**(95% CI)**	**P**_**hetr**_	**P**_**EM**_
Overall	11	-0.96 (-1.52, -0.40)	80 (65, 89)	<0.001		8	-2.19 (-3.19, -1.19)	71 (40, 86)	0.001	
Study design										
Parallel	5	-0.78 (-1.93, 0.37)	86 (69, 94)	<0.001	0.48	4	-1.71 (-2.73, -0.70)	59 (0, 86)	0.07	0.33
Crossover	6	-1.20 (-2.03, -0.38)	77 (49, 90)	0.01		4	-3.32 (-5.90, -0.75)	82 (54, 93)	0.001	
Region										
India	7	-1.43 (-2.26, -0.60)	48 (0, 78)	0.07	0.21	6	-2.60 (-4.06, -1.13)	73 (39, 88)	0.002	0.50
Others	4	-0.55 (-1.25, 0.16)	89 (75, 95)	<0.001		2	-1.59 (-3.08, -0.10)	74 (0, 94)		
Study precision^a^										
Below median	5	-0.54 (-1.15, 0.07)	86 (69, 94)	<0.001	0.10	4	-1.55 (-2.52, -0.57)	67 (2, 89)	0.03	0.11
Above median	6	-1.68 (-2.62, -0.74)	43 (0, 77)	0.12		4	-3.42 (-5.56, -1.28)	67 (4, 89)	0.03	
Study population^b^										
Healthy	2	0.05 (-0.51, 0.61)	82 (23, 96)	0.02		-	-	-	-	
T1DM	1	-4.20 (-6.73, -1.67)	-	-	0.01	1	-3.20 (-6.92, 0.52)	-	-	0.79
T2DM	8	-1.21 (-1.69, -0.73)	26 (0, 67)	0.22		7	-2.14 (-3.19, -1.09)	74 (45, 88)	0.001	
Randomization										
Yes	9	-0.94 (-1.56, -0.32)	83 (69, 91)	<0.001	0.83	6	-2.54 (-3.87, -1.21)	73 (37, 88)	0.003	0.49
Unknown	2	-1.11 (-2.10, -0.12)	0 (0, 100)	0.49		2	-1.58 (-3.61, 0.45)	81 (17, 95)	0.02	
Blinding status										
Double-blinded	4	-0.44 (-1.22, 0.34)	88 (72, 95)	<0.001	0.15	2	-1.95 (-3.02, -0.88)	16 (0, 55)	0.28	0.74
Unknown	7	-1.37 (-2.08, -0.67)	47 (0, 78)	0.08		6	-2.43 (-3.82, -1.04)	78 (50, 90)	0.000	
Daily dose (grams)^b^										
<5	3	0.02 (-0.46, 0.50)	63 (0, 89)	0.07		1	-1.34 (-2.83, 0.14)	-	-	
5-10	4	-1.27 (-1.80, -0.74)	21 (0, 88)	0.28	0.01	4	-1.55 (-2.52, -0.57)	67 (2, 89)	0.04	0.02
>10	4	-2.07 (-3.58, -0.55)	68 (9, 89)	0.02		3	-4.42 (-5.96, -2.89)	8 (0, 35)	0.58	
Preparation^b^										
Powdered seed	5	-1.12 (-1.63, -0.61)	29 (0, 72)	0.23		4	-1.55 (-2.52, -0.57)	67 (2, 89)	0.03	
Hydro-alcoholic extract	3	0.02 (-0.46, 0.50)	63 (0, 89)	0.07	0.002	1	-1.34 (-2.83, 0.14)	-	-	0.05
Debitterized seed powder	3	-2.68 (-3.82, -1.54)	0 (0, 90)	0.42		3	-4.42 (-5.96, -2.89)	0 (0, 90)	0.58	
Study duration (days)										
<30	5	-1.48 (-2.64, -0.32)	80 (52, 91)	0.001	0.39	3	-4.42 (-5.96, -2.89)	8 (0, 35)	0.58	0.02
> = 30	6	-0.78 (-1.68, 0.11)	83 (65, 92)	<0.001		5	-1.49 (-2.28, -0.70)	55 (0, 84)	0.06	
Mean Age (years)										
<43.1	4	-0.73 (-1.61, 0.16)	86 (67, 94)	<0.001		2	-4.14 (-5.80, -2.47)	0 (0, 100)	0.58	
> = 43.1	5	-1.11 (-1.76, -0.47)	45 (0, 80)	0.12	0.60	4	-1.90 (-3.19, -0.61)	67 (4, 89)	0.03	0.21
Mean BMI (kg/m^2^)										
<25	3	-1.62 (-3.21, -0.02)	93 (82, 97)	<0.001	0.29	2	-2.52 (-3.75, -1.30)	0 (0, 100)	0.71	0.17
> = 25	3	-0.19 (-1.14, 0.76)	74 (12, 92)	0.02		2	-1.01 (-1.72, -0.30)	0 (0, 100)	0.62	

*Abbreviations*: *BMI* body mass index, *CI* confidence interval, *P*_*hetr*_ p value for heterogeneity, *P*_*EM*_ p value for effect modification.

^a^The cutoffs are based on the median of standard error of the effect size for fasting blood glucose and 2hr glucose respectively; For mean age and study duration, the cutoffs are based on the median values of 11 data points.

^b^P_EM_-value for the overall F-test for a common mean amongst the three groups (i.e., study population, and fenugreek drug preparation format), the overall p-value for daily dose was obtained by modeling this as a continuous variable in meta-regression analysis.

The funnel plot for effects of fenugreek on fasting blood glucose by study precision appeared to be asymmetrical (Additional file 1: Figure S1, Begg test, p = 0.10) and the Egger test was significant (p = 0.03) suggesting potential publication bias. However, these tests are based on detecting an association between study precision (lower SE of effect estimates) and effect size. Less precise studies also tended to use a greater dose of fenugreek (r = 0.51 between dose and SE of the effect estimates) and we could therefore not distinguish between potential publication bias and the dose of fenugreek used.

#### 2 hr glucose

**Figure 3:**
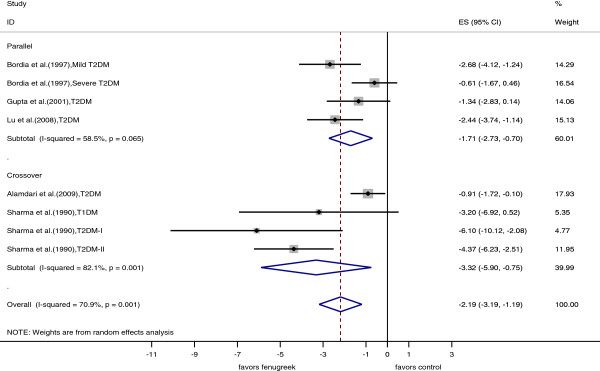
**Forest plot of the effect of fenugreek on 2 hour postload glucose.** The effects in individual trials are depicted as open squares with 95% confidence intervals (CIs). Pooled estimates with 95% CIs are depicted as open diamonds.

Seven trials (8 data points) reported effects of fenugreek on 2 hr glucose values. Forest plots of 2 hr glucose effects in individual trials and the pooled analyses are shown in Figure 3. Meta-analysis of the trials yielded a pooled estimate for the effect of fenugreek on 2 hr glucose of -2.19 mmol/l (95% CI: -3.19, -1.19, p <0.001). There was large heterogeneity in study results (I^2^ = 71%; p = 0.001). Stratified analyses of effects of fenugreek on 2 hr glucose according to study characteristics are presented in Table 2. As observed for fasting glucose, a higher dose was associated with greater effects on 2 hr glucose concentrations. We also observed stronger effects for trials with study duration less than 30 days than for trials with a longer duration and for trials that administered debitterized fenugreek seed powder than for trials using other fenugreek preparations. However, the trials of short duration that used debitterized fenugreek powder were the same three trials that administered a substantially higher dose of fenugreek than the other trials. Heterogeneity in effects of fenugreek on 2 hr glucose was partly explained by fenugreek dose (I^2^_residual_ = 42%; adjusted R^2^ = 72%) and fenugreek preparation method (I^2^_residual_ = 50%; adjusted R^2^ = 61%). The funnel plot for 2 hr glucose data appeared to be asymmetrical (Additional file 1: Figure S2, Begg test, p = 0.05) and the Egger test (p = 0.03) also provided evidence for publication bias. Again, less precise studies also tended to use a greater dose of fenugreek (r = 0.54 between dose and SE of the effect estimates) and we could not distinguish between potential publication bias and the dose of fenugreek used.

#### Other outcome measures (HbA1c and fasting serum insulin)

Three trials (2 parallel and 1 crossover trial) reported the effects of fenugreek on HbA1c. Fenugreek significantly reduced HbA1c values as compared with control treatment (pooled mean difference -0.85%; 95% CI: -1.49%, -0.22%, p = 0.009) (Additional file 1: Figure S3) without significant heterogeneity in study results (I^2^ = 0%; p = 0.78).

Five trials reported fasting serum insulin concentrations. However, fasting insulin may have been affected by exogenous insulin use in persons with type 1 diabetes and fasting insulin values had a strongly skewed distribution in persons with type 2 diabetes. Therefore, we only included two trials [25,43] in persons without diabetes in our meta-analysis of the effects of fenugreek on fasting insulin. The pooled effect of fenugreek on fasting serum insulin was not statistically significant (pooled mean difference = -1.42 mU/L; 95% CI: -3.04, 0.19 mU/L; p = 0.08) (Additional file 1: Figure S4). Heterogeneity for effects on fasting insulin was substantial, but not statistically significant (I^2^ = 62%; p = 0.10). Subgroup analyses were not performed for HbA1c and fasting serum insulin because of the limited number of trials for these outcomes.

### Sensitivity analyses

We conducted a sensitivity analysis excluding the trial that did not report randomization status. This exclusion had little effect on the pooled effect on fasting blood glucose (-0.94; 95% CI: -1.56, -0.32) or 2 hr glucose (-2.54; 95% CI: -3.87, -1.21). We also conducted sensitivity analyses for the effects of fenugreek on fasting blood glucose and 2 hr glucose concentrations omitting one study at a time. None of the individual trials dramatically influenced pooled effect estimates, which ranged from -0.78 mmol/l (95% CI: -1.31, -0.25) to -1.20 (95% CI: -1.85 to -0.56) for fasting blood glucose and from -1.79 mmol/l (95% CI: -2.69, -0.90) to -2.52 (95% CI: -3.64 to -1.39) for 2 hr glucose. We also conducted sensitivity analyses with simultaneous exclusion of trials conducted by the same researchers. The three trials reported by Sharma et al. [22,44] had a much larger dose of fenugreek than the other trials and also the lowest precision. In a sensitivity analysis that excluded these trials, the pooled effect on fasting blood glucose (-0.62 mmol/l, 95% CI: -1.14, -0.10) and 2 h glucose (-1.49 mmol/l, 95% CI: -2.28, -0.70) remained statistically significant. We also conducted a sensitivity analysis after excluding the Chevassus trials ([25,43]) that were the only trials in persons without diabetes, used a low dose, and had the largest precision. After exclusion of these studies, the pooled effect estimate for fasting glucose was larger (-1.35 mmol/L; 95% CI: -1.92, -0.74).

### Adverse effects

Chevassus et al. reported 2 cases of specific urine smell and 1 case of abdominal pain in one trial [25] and 4 cases of mild gastrointestinal symptoms, and 1 case of specific urine and sweat smell in both the treatment group and the control group in their other trial [43]. Of the 12 participants in the treatment group in the study by Gupta et al. [23], 5 developed dyspepsia and mild abdominal distention for the first few days of therapy. This subsided on continuation of the therapy. No renal or hepatic side effects were reported and there were no withdrawals due to the side effects. Lu et al. [9] reported that 2 out of 46 participants of the treated group suffered from stomach discomfort and nausea, and one from diarrhea during the treatment period. These symptoms disappeared after 2 day drug withdrawal without special treatment. No adverse reaction was found after the treatment resumed and all participants finished the trial.

## Discussion

In our meta-analysis of 10 clinical trials, intake of fenugreek seeds resulted in a significant reduction in fasting blood glucose, 2 hr glucose, and HbA1c. However, we observed substantial heterogeneity in study results. Differences in the diabetes status of participants and the large variation in dose of fenugreek seed extract used and type of preparation appeared to be contributors to variation in study results. No major harmful side effects of fenugreek were reported in all included studies.

We only found a significant reduction in glucose parameters for trials that administered medium to high doses (≥5 g) of fenugreek seed powder and not for trials that administered low doses (< 2 g) of hydro-alcoholic extracts. Medium to high doses (range: 5–25 g) of fenugreek seed powder also lowered postprandial glucose levels in acute studies [6,18,19,21,45,46]. Lower doses, as used in three of the trials in our meta-analyses, were not evaluated in acute studies of fenugreek.

The mechanisms by which fenugreek may lower blood glucose levels have not been well established in humans. Acute hypoglycemic effects of fenugreek seeds and its extract have been evaluated in individuals with and without diabetes [18,22,44]. Whole fenugreek raw seeds, extracted seed powder, cooked seeds (25 g) and gum isolate of seeds (5 g) decreased postprandial glucose levels, whereas degummed seeds (25 g) showed little effect [18]. These findings suggest that acute effects of fenugreek seeds are mainly due to the gum fraction, but do not exclude a longer term effect of other fenugreek components on glycemia. Animal studies also indicate that the soluble fiber fraction of fenugreek seeds reduces the rate of enzymatic digestion and the absorption of glucose from the gastrointestinal tract [8]. However, data from other studies suggest an effect of other fenugreek components on glucose homeostasis. In diabetic rats, trigonelline ingestion increased insulin sensitivity and reduced blood glucose levels [47]. In addition, a novel amino acid derivative extracted from fenugreek seeds, 4-hydroxyisoleucine, stimulated glucose-dependent insulin release in isolated rat and human pancreatic islet cells [14]. In a trial of acute effects in healthy volunteers, trigonelline reduced the early glucose response during an OGTT [48].

The only previous meta-analysis of the effects of fenugreek on glycemia included only two clinical trials as compared with 10 in the current meta-analysis [26]. Strengths of our study included the comprehensive literature search leading to the identification of a reasonably large number of trials and a detailed analysis of potential sources of heterogeneity in study results. Our study also has several limitations that need to be considered in the interpretation of the results. First, the quality of the included trials was generally poor. None of the trials reported the methods of randomization or allocation concealment, and only a few trials provided information on blinding status and drop-out rates. In addition, with some exceptions [9] it was unclear whether other diabetes medication remained constant during the trial. Most of the included crossover trials did not test the carryover effect or report a washout period. However, we did not find a difference in results between parallel and cross-over trials suggesting that carryover effects did not substantially affect the results. Second, tests for publication bias suggested that such bias may have been present. Tests for publication bias are based on detecting differences in effect sizes by study precision with a greater effect size for less precise ('smaller’) studies suggesting the presence of publication bias. In our meta-analysis less precise studies were also more likely to use larger doses of fenugreek. Differences in dose are thus a possible alternative explanation for the observed 'small study effect’, but we were unable to distinguish between the effects of dose and publication bias on effect sizes. Finally, we only found a significant effect on glycemia for powdered fenugreek seeds and our findings do not apply to other forms of fenugreek and may differ for other strains as a result of natural variation in active ingredients.

Our systematic review and meta-analysis suggest that fenugreek seeds may contribute to better glycemic control in persons with diabetes mellitus with a similar magnitude of effect as intensive lifestyle [49] or other pharmaceutical treatment added to standard treatment [50]. Fenugreek is widely available at low cost and generally accepted in resource poor countries such as India and China where a large proportion of persons with diabetes in the world reside. Therefore, fenugreek may be a promising complementary option for the clinical management of diabetes. The previously reported lipid lowering effect of fenugreek may be an additional benefit [19,44,51]. However, given the limited quality of the included trials and potential for publication bias, a larger double blind randomized trial should be conducted according to rigorous standards for herbal interventions [31] with an appropriate randomization procedure, an adequate method of allocation concealment and transparent reporting of these methods. The fenugreek herbal product must be standardized and tested for the composition and can be administered in the form of capsules with a recommended dose of at least 5 g per day. In order to provide more conclusive evidence on the benefit of fenugreek for glucose homeostasis, a trial in at least 100 (50 subjects in each of the study arms) persons with diabetes is warranted. The duration should preferably be at least three months to be able to evaluate effects on HbA1c levels and given the longer duration a parallel trial appears most appropriate.

## Competing interest

The authors declare that they have no competing interest.

## Authors’ contributions

NN, MN and RMvD contributed to the conception and design of the study. NN and MN conducted the literature search and data extraction. NN performed the statistical analyses. NN and MN drafted the manuscript. RMvD supervised the study. NN, RJD and RMvD contributed to the interpretation of data and critically revised the manuscript for important intellectual content. All authors gave final approval. NN and RMvD are the guarantors of this work and, as such, had full access to all the data in the study and take responsibility for the integrity of the data and the accuracy of the data analysis.

## Supplementary Material

Additional file 1: Figure S1 Funnel plot for effect of fenugreek on fasting blood glucose. The solid line represents the pooled effect estimate expressed as the weighted mean difference and the dashed lines represent pseudo-95% confidence limits. **Figure S2.** Funnel plot for effect of fenugreek on 2 hour postload glucose. The solid line represents the pooled effect estimate expressed as the weighted mean difference and the dashed lines represent pseudo-95% confidence limits. **Figure S3.** Forest plot of the effect of fenugreek on HbA1c. The effects in individual trials are depicted as open squares with 95% confidence intervals (CIs). The pooled estimate with 95% CI is depicted as an open diamond. **Figure S4.** Forest plot of the effect of fenugreek on fasting serum insulin. The effects in individual trials are depicted as open squares with 95% confidence intervals (CIs). Pooled estimate with 95% CI is depicted as an open diamond. Click here for file
