# Supplementary material for: Effect of fenugreek (Trigonella foenum-graecum L.) intake on glycemia: a meta-analysis of clinical trials
Source: Nutr J. 2014 Jan 18;13:7. doi: 10.1186/1475-2891-13-7 (PMC3901758; doi:10.1186/1475-2891-13-7)
Supplement: Additional file 1: Figure S1 — Funnel plot for effect of fenugreek on fasting blood glucose. The solid line represents the pooled effect estimate expressed as the weighted mean difference and the dashed lines represent pseudo-95% confidence limits. Figure S2. Funnel plot for effect of fenugreek on 2 hour postload glucose. The solid line represents the pooled effect estimate expressed as the weighted mean difference and the dashed lines represent pseudo-95% confidence limits. Figure S3. Forest plot of the effect of fenugreek on HbA1c. The effects in individual trials are depicted as open squares with 95% confidence intervals (CIs). The pooled estimate with 95% CI is depicted as an open diamond. Figure S4. Forest plot of the effect of fenugreek on fasting serum insulin. The effects in individual trials are depicted as open squares with 95% confidence intervals (CIs). Pooled estimate with 95% CI is depicted as an open diamond. [file 1475-2891-13-7-S1.doc]

***Additional file 1: Figure S1*: Funnel plot for effect of fenugreek on fasting blood glucose**

The solid line represents the pooled effect estimate expressed as the weighted mean difference and the dashed lines represent pseudo-95% confidence limits.

***Additional file 1: Figure S2*: Funnel plot for effect of fenugreek on 2 hour postload glucose**

The solid line represents the pooled effect estimate expressed as the weighted mean difference and the dashed lines represent pseudo-95% confidence limits.

***Additional file 1: Figure S3*: Forest plot of the effect of fenugreek on HbA1c**

The effects in individual trials are depicted as open squares with 95% confidence intervals (CIs). The pooled estimate with 95% CI is depicted as an open diamond.

***Additional file 1: Figure S4*: Forest plot of the effect of fenugreek on fasting serum insulin**

The effects in individual trials are depicted as open squares with 95% confidence intervals (CIs). Pooled estimate with 95% CI is depicted as an open diamond.
